# An Elusive Diagnosis: Delays in Treatment and Opportunities for Improvement in Temporal Encephalocele and CSF Leak

**DOI:** 10.1097/ONO.0000000000000026

**Published:** 2023-01-12

**Authors:** Steven D. Curry, Colin E. McCorkle, Jonathan L. Hatch, Geoffrey C. Casazza

**Affiliations:** 1 Department of Otolaryngology-Head and Neck Surgery, University of Nebraska Medical Center, Omaha, NE.

**Keywords:** Cerebrospinal fluid leak, Encephalocele, Skull base surgery

## Abstract

**Objective:**

Symptoms of temporal encephalocele or cerebrospinal fluid (CSF) leak causing middle ear effusion or otorrhea can be nonspecific and mistaken for other common diagnoses, leading to delays in diagnosis, failed treatments, and a risk of meningitis. This study sought to investigate the association between symptomatology and time to definitive surgical management.

**Study Design:**

Retrospective cohort.

**Setting:**

Single tertiary care academic medical center.

**Patients:**

Adults treated surgically for temporal encephalocele or CSF leak. Revision cases were excluded.

**Interventions:**

Chart review was performed to identify pertinent symptoms at presentation. Multivariable regression was performed to analyze the association between symptoms and time to definitive management.

**Main Outcome Measures:**

Otologic and related symptoms present prior to middle cranial fossa (MCF). Time between symptom onset and surgical treatment.

**Results:**

Thirty-four patients had symptoms present a median of 15.5 months (interquartile range, 8–35 months; range, 1 month to 12 years) prior to surgery. The most common symptoms were subjective hearing loss in the affected ear (76.5%) and aural fullness (73.5%). Otorrhea was present in 55.9%, and 42.9% had a history of otorrhea after myringotomy with or without tube insertion. Meningitis occurred in 5 patients (14.7%). Only the absence of otalgia was statistically significantly associated with decreased time between symptoms onset and surgery (*P* = 0.01).

**Conclusions:**

Encephalocele and CSF leak were most commonly associated with aural fullness and hearing loss. Medical treatment for presumed Eustachian tube dysfunction or chronic ear disease were commonly observed. Patients had symptoms for a median of almost 1 and a half years prior to surgical management.

Spontaneous cerebrospinal fluid (CSF) leak and temporal bone encephaloceles can occur from the lateral skull base in patients with dehiscences of the tegmen tympani and/or the tegmen mastoideum, which produce a communication between the pneumatized skull base and the meninges. There has been an increase in the rate of craniotomy for CSF leak, with doubling of the rate of craniotomy for CSF leak from 2002 to 2012 in the United States, associated with increasing obesity rates (1,2). While the causes of the increased rates of CSF leak and/or encephalocele are not entirely clear, there is an association between spontaneous CSF leak and obesity, idiopathic intracranial hypertension, and obstructive sleep apnea due to the elevated intracranial pressure in patients with these conditions (3,4).

Symptoms of lateral skull base CSF leak or temporal encephalocele are often nonspecific and commonly include middle ear effusion, aural fullness, and ipsilateral conductive hearing loss. Otorrhea can occur in the setting of a tympanic membrane perforation or after myringotomy and tympanostomy tube insertion. Signs and symptoms of these pathologies can be easily mistaken for more common diagnoses such as eustachian tube dysfunction or chronic otitis media. More recently, headache, pulsatile tinnitus, and dizziness/vertigo have become recognized as symptoms occurring with CSF leak in the temporal bone (5–7). Diagnosis can be challenging, and patients with spontaneous CSF leak often have a protracted length of time between onset of symptoms and diagnosis, with a reported mean delay of 35.4 months reported in 1 series (8), and some patients having symptoms for over a decade (9). While some of these symptoms may be merely a nuisance, a persistent CSF fistula provides a route for an ascending pathogen, which places the patient at risk for intracranial infections such as meningitis or an intracranial abscess (10). Bacterial meningitis can lead to mortality or severe morbidity including seizures, encephalopathy, or cranial nerve deficits despite prompt treatment with antibiotics (11).

This study sought to investigate the association between the symptomatology of lateral skull base CSF leak or encephalocele and the duration from identification of symptoms to definitive surgical management in order to identify how the presentation of the disease impacted delays in definitive management. We hypothesized that patients with nonspecific otologic symptoms, such as aural fullness, or who seemed to fit a pattern consistent with other etiologies would have a prolonged time course prior to definitive treatment.

## METHODS

A retrospective chart review was performed for all adult patients diagnosed with temporal encephalocele or CSF leak who underwent surgical repair from January 2018 to August 2021 at the University of Nebraska Medical Center. Current Procedural Terminology codes for middle cranial fossa (MCF) skull base procedures (61590 and 61591), and International Classification of Diseases, 10th Revision codes Q01.8 (encephalocele of other sites), Q01.9 (encephalocele, unspecified), and G96.0 (CSF leak) were used to identify patients from the electronic medical record. Patient charts were reviewed to verify diagnoses. Revision cases and patients who underwent additional concurrent operations or surgery without the operative indication being CSF leak or encephalocele (such as traumatic, iatrogenic, or tumor-related CSF leak) were excluded.

Chart review was performed to identify pertinent symptoms at clinical presentation and any pre-surgical follow-up visits in primary care or otolaryngology clinics. The beginning of symptoms was defined by patient-reported onset as recorded in the medical record. Four patients who had symptoms for an indeterminate time period reported as “several” or “many” years were coded as having symptoms for 3 years for quantitative analysis. Demographic and clinical data including sex, age, race, ethnicity, body mass index (BMI), medical and surgical history, radiographic findings, beta-2 transferrin testing results, and the method of surgical repair were collected. The main outcome measure was the length of time between onset of otologic and related symptoms and surgical treatment.

Data were summarized with descriptive statistics. Continuous variables were reported as mean and SD if normally distributed or median and interquartile range (IQR) if not normally distributed. Normality was determined using the Shapiro-Wilk test. Categorical variables were summarized as percentages and analyzed with Fisher exact test. Multivariable regression was performed to identify the independent association between presenting symptoms and decreased time from symptom onset to definitive surgical repair. Variation inflation factor was calculated to assess for collinearity of factors in the regression analysis.

Statistical analysis was performed in R version 4.1.2 (R Foundation for Statistical Computing, Vienna, Austria). An α = 0.05 was used to determine statistical significance for all statistical tests. Institutional review board (IRB) approval was obtained from the University of Nebraska Medical Center (IRB No. 412-19-EX).

## RESULTS

There were 34 patients identified who underwent operative repair of lateral skull base CSF leak and/or temporal encephalocele. The mean age was 53.7 ± 11.7 years. Twenty-two (64.7%) of the patients were female. Median BMI was 37.5 kg/m^2^, with 67.6% being obese. Baseline demographic and clinical data are shown in Table 1 . All surgeries were performed using a team approach with a both neurotologist and a skull base neurosurgeon working together.

**TABLE 1.** Patient demographics and baseline characteristics

Variable	Overall	CSF leak	Encephalocele	CSF leak and encephalocele	*P*
Sex, n (%)					0.89^*a*^
Male, n (%)	12 (35.3)	1 (20.0)	4 (36.4)	7 (38.9)	
Female	22 (65.7)	4 (80.0)	7 (63.6)	11 (61.1)	
Age, mean ± SD	53.7 ± 11.7	52.5 ± 12.2	51.7 ± 8.1	55.2 ± 13.3	0.15^*b*^
BMI, mean ± SD	37.5 ± 10.5	40.1 ± 15.7	32.8 ± 6.1	39.6 ± 10.5	0.20^*b*^
Race, n (%)					0.71^*a*^
White	29 (85.3)	5 (100.0)	9 (81.8)	15 (83.3)	
Black	4 (11.8)	0 (0.0)	1 (9.1)	3 (16.7)	
Other	1 (2.9)	0 (0.0)	1 (9.1)	0 (0.0)	
Ethnicity, n (%)					0.21^*a*^
Hispanic	2 (5.9)	0 (0.0)	2 (18.2)	0 (0.0)	
Non-Hispanic	32 (94.1)	5 (100.0)	9 (81.8)	18 (100.0)	
Time to diagnosis, months (IQR)	15.5 (8–35)	11 (9–27)	12 (7–30)	18 (10–35)	0.80^*a*^
Beta-2 transferring testing					
Positive	15	3	1	11	**0.01**
Negative	4	0	1	3	
Not tested	15	2	9	4	
Surgical approach, n (%)					
MCF alone	12 (35.3)	2 (40.0)	7 (63.6)	3 (16.7)	**0.03** ^***a***^
MCF-mastoidectomy	22 (64.7)	3 (60.0)	4 (36.4)	15 (83.3)	

Percentages are out of column totals. Bold values denote statistical significance.

^*a*^Fisher exact test between CSF leak, encephalocele, and CSF leak and encephalocele groups.

^*b*^ANOVA between CSF leak, encephalocele, and CSF leak and encephalocele groups.

BMI indicates body mass index; CSF, cerebrospinal fluid; IQR, interquartile range; MCF, middle cranial fossa; n, number.

**FIG. 1.**
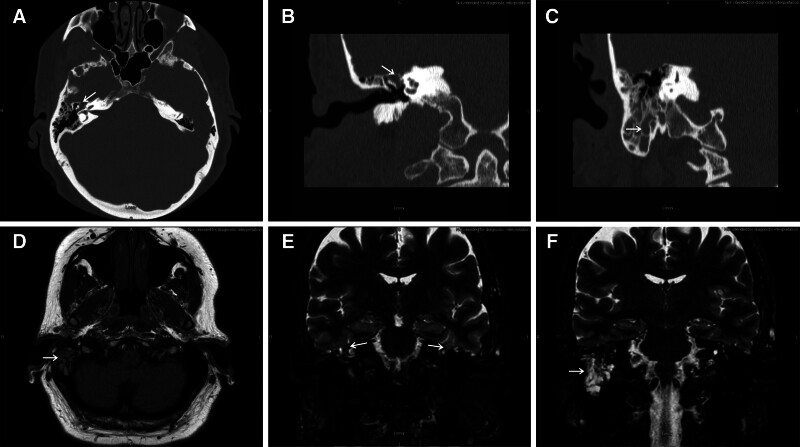
Bilateral temporal encephaloceles. Computed tomography (CT) (*A*–*C*) and MRI (*D*–*F*) images of a patient with bilateral temporal encephaloceles and right mastoid effusion. *A*, Axial CT showing right encephalocele between the cochlea and ossicular chain (arrow). *B*, Coronal CT showing right encephalocele between the cochlea and ossicular chain (arrow). *C*, Coronal CT posterior to the previous image showing mastoid effusion (arrow). *D*, Axial T-1 weighted MRI image showing right mastoid effusion (arrow). *E*, Coronal T-2 weighted MRI showing bilateral temporal encephaloceles (arrows). *F*, Coronal T-2 weighted MRI image showing right middle ear effusion (arrow).

Patients had symptoms present for a median of 15.5 months (IQR, 8–35 months; mean, 23.1 ± 25.8 months) prior to definitive surgical treatment. No difference in time to treatment was found between patients with CSF leak alone, encephalocele alone, or both CSF leak and encephalocele (*P* = 0.80). The most common symptoms were subjective hearing loss in the affected ear (76.5%) and aural fullness (73.5%). CSF otorrhea was present in 55.9%, and 41.2% had a history of myringotomy with or without tympanostomy tube insertion. Meningitis occurred in 5 patients (14.7%) prior to surgery. Table 2 shows the presenting symptoms. Figure 1 shows representative imaging findings in a patient with bilateral temporal encephaloceles with a right mastoid effusion due to a CSF leak.

**TABLE 2.** Presenting symptoms and multivariable regression

Symptom	n (%)^*a*^	Coefficient	SE	*P*
Aural fullness	25 (73.5)	20.0	18.3	0.2
Otalgia	8 (23.5)	38.4	15.0	**0.01**
Hearing loss	26 (76.5)	1.3	13.6	0.93
Pulsatile tinnitus	0 (0.0)	—	—	—
Nonpulsatile tinnitus	10 (29.4)	–5.8	12.4	0.65
CSF otorrhea	19 (55.9)	–18.3	15.5	0.25
CSF rhinorrhea	4 (11.8)	24.2	15.4	0.13
Otorrhea after tympanostomy tube	14 (41.2)	–2.6	17.7	0.88
Meningitis	5 (14.7)	–10.7	18.5	0.57
Brain abscess	1 (2.9)	17.2	41.5	0.68
Chronic otitis media/mastoiditis	8 (23.5)	9.5	11.9	0.43
Headache	12 (35.3)	–16.2	13.4	0.24
Vision changes	2 (5.9)	24.6	25.8	0.35
Seizure	2 (5.9)	–15.3	32.4	0.64

Values were not calculated for pulsatile tinnitus because no patients presented with this symptom. Bold values denote statistical significance.

^*a*^Percentages calculated out of total number of patients.

— indicates Not applicable.

CSF indicates cerebrospinal fluid; n, number.

In the multiple regression analysis, only the absence of otalgia was statistically significantly associated with time between symptoms onset and surgery, with the presence of otalgia being associated with a longer period of time from symptom onset to operative intervention (*P* = 0.01) (Table 2). No collinearity was observed between factors. The overall model using all of the symptoms assessed was not predictive of the variation in time from symptom onset to surgery (adjusted *R*^2^ = 0.10; *P* = 0.30).

## DISCUSSION

Patients with CSF leak or encephalocele who underwent operative repair most commonly had nonspecific otologic symptoms of aural fullness and hearing loss. Medical treatment for presumed Eustachian tube dysfunction or myringotomy with subsequent CSF otorrhea in the ipsilateral ear were commonly observed. Patients had symptoms for a median of almost 1 and a half years prior to definitive surgical management.

The absence of otalgia was the only factor that was statistically significantly associated with decreased time from symptom onset to operative repair. Pain inconsistent with physical examination or pain refractory to medical management could prompt physicians to obtain imaging that can identify a defect in the tegmen; however, patients presenting with pain that is attributed to infectious or inflammatory etiologies may not undergo imaging if it is not thought to be useful for diagnostic evaluation, or if imaging with Computed tomography (CT) were obtained, the fluid/soft tissue density in the middle ear and/or mastoid may be misattributed to etiologies other than CSF leak or encephalocele. Low rates of otalgia reported for patients with CSF leak or encephalocele, together with the relative rarity of these clinical entities illustrate the need to have a high index of suspicion in treating atypical cases of middle ear effusion or otorrhea (5–7,12,13).

Patient presentation and demographics were consistent with the published literature. Lateral skull base CSF leak or encephalocele was more common in patients with elevated BMI and female sex (6,14). Persistent unilateral clear otorrhea, such as following myringotomy with or without tympanostomy tube insertion, was common among our patients (15). Most patients had CSF otorrhea, with almost half of the patients having CSF otorrhea after myringotomy and insertion of a tympanostomy tube. The average BMI among the CSF leak alone, encephalocele, and combined CSF leak and encephalocele groups were all about the threshold for a diagnosis of obesity.

The rate of meningitis of 15% among our sample may overestimate the natural history of meningitis in patients with spontaneous CSF otorrhea as some patients with intermittent CSF leak may never ultimately undergo surgical management. Our rate of meningitis is similar to the rate of a previously published series, which reported 4 out of 20 patients (20%) treated for surgically confirmed temporal bone dehiscence leading to encephalocele and/or CSF otorrhea had presented with meningitis and/or intracerebral abscess (16). Among our sample, 4 out of 5 patients with meningitis were diagnosed with both a CSF leak and an encephalocele, with the 5th patient having an encephalocele but no diagnosed CSF leak. Only 1 patient had prior otologic symptoms. She had been diagnosed with a unilateral middle ear effusion that was attributed to eustachian tube dysfunction.

Laboratory testing of otorrhea specimen can be performed to confirm the presence of CSF-specific proteins in a patient suspected of having a CSF leak. Testing using a beta-2 transferrin assay is a reliable method for confirming suspected CSF leak and has been recommended as the primary method for screening or confirming the diagnosis (17,18). Among our sample, statistically significant differences were found between diagnosis groups for beta-2 transferrin results, with most patients with CSF leak alone or CSF leak and encephalocele testing positive for beta-2 transferring, and most patients with encephalocele alone not being tested. Despite high sensitivity and specificity of the assay, there was not perfect concordance between test results and diagnosis among our sample. There were 3 patients with CSF leak at the time of surgery testing negative previously. One patient with encephalocele but no CSF leak at the time of surgery previously tested positive for beta-2 transferrin. Point-of-care beta-trace protein assay is an emerging testing option to diagnose rapidly diagnose CSF leak (19,20). Persistent clear otorrhea that is pulsatile or not responsive to medical management should prompt consideration of CSF leak in the differential diagnosis.

CT and MRI provide complimentary information for diagnosis and surgical planning. High resolution CT is the preferred imaging modality to identify the location and size of an osseous dehiscence in the floor of the middle fossa (21–23). MRI can be used to differentiate between encephalocele and chronic otitis or cholesteatoma (21,24,25). Signal intensity of fluid on MRI with 3D heavily-T2 weighted sequences (eg, fast imaging employing steady-state acquisition or constructive interference in steady state) and fluid-attenuated inversion recovery sequences have high sensitivity and specificity for distinguishing CSF leaks from other causes of middle ear effusion (26,27). In patients with unclear diagnoses and intact tympanic membranes who are unwilling to undergo myringotomy or tympanocentesis for middle ear fluid sampling, or who have intermittent CSF leak and are unable to collect sufficient fluid for laboratory analysis, imaging studies can help to rule-in or -out a diagnosis of CSF leak or encephalocele.

This study is limited by data present in the medical record due to the retrospective design. One limitation of our analysis was the need to interpret an unclear duration of symptoms in 4 out of 34 patients who reportedly had symptoms for “several” or “many” years. For the purpose of quantitative analysis, these patients were coded as having symptoms for 3 years. This was chosen in order to not bias the results in a way that would significantly increase the average time from onset of symptoms to operative management, but it is likely a conservative estimate. While patient charts were thoroughly reviewed to identify presenting symptoms, clinical notes do not necessarily record the presence or absence of all pertinent symptoms that could be identified using a checklist in a prospective collection of data.

Overall, patients had a prolonged period between onset of symptoms and definitive management with operative repair of the skull base using a MCF or combined MCF-mastoidectomy approach. Patient symptomology was not significantly associated with the time from symptom onset to surgery and only accounted for a small amount of the variation in time between symptom onset and operative repair among patients.

## FUNDING SOURCES

None declared.

## CONFLICT OF INTEREST

None declared.

## DATA AVAILABILITY STATEMENT

The datasets generated during and/or analyzed during the current study are not publicly available, but are available from the corresponding author on reasonable request.
